# Coordination Elicits Synchronous Brain Activity Between Co-actors: Frequency Ratio Matters

**DOI:** 10.3389/fnins.2019.01071

**Published:** 2019-10-15

**Authors:** Xiaojun Cheng, Yafeng Pan, Yinying Hu, Yi Hu

**Affiliations:** ^1^School of Psychology, Shenzhen University, Shenzhen, China; ^2^School of Psychology and Cognitive Science, East China Normal University, Shanghai, China

**Keywords:** interpersonal brain synchronization, interpersonal coordination, multifrequency pattern, fNIRS-based hyperscanning, frontopolar

## Abstract

People could behave in two different ways when engaging in interpersonal coordination activities: moving at the same frequency (isofrequency pattern, IP; the movement frequency ratio is 1:1) or at different frequencies (multifrequency pattern, MP; the movement frequency ratio is non 1:1). However, how the interpersonal coordination pattern modulates coordination outcome and the related brain-to-brain connectivity is not fully understood. Here, we adopted a continuous joint drawing task in which two participants co-drew parallelogram shapes according to two coordination patterns (i.e., IP vs. MP) while their brain activities were simultaneously recorded by the functional near-infrared spectroscopy (fNIRS) based hyperscanning technique. Dyads showed better coordination performance, as well as relatively greater interpersonal brain synchronization (IBS) at the left frontopolar area, in the MP condition compared to the IP condition. Granger causality analyses further disclosed the bidirectional influences between the brains of the coordinating individuals. Such interpersonal influences were enhanced when individuals coordinated in the MP condition. Finally, the IBS during coordination was related to the dyadic self-control level. Taken together, our study revealed that interpersonal multifrequency coordination pattern facilitates the coordination efficiency, which was associated with the enhanced brain-to-brain connectivity. Our work also suggests the potentially positive role of self-control during the interpersonal coordination process.

## Introduction

People frequently engaged in social interactions, the achievement of which are largely relied on our capacity to coordinate behaviors with others in space and time (Sebanz et al., 2006; Richardson et al., 2007; Nessler and Gilliland, 2009). In some coordination activities, we just perform movements at the same frequency (i.e., *isofrequency pattern, IP*), or mirroring, such as side-by-side walking or together singing/humming. In some other activities, however, we are required to perform non-mirroring pattern—acting at different frequencies (i.e., *multifrequency pattern, MP)*, such as dancing or sports. Various interpersonal coordination tasks based on either MP or IP are developed and studied for different researching purposes separately. However, in real life people sometimes need to balance different coordination strategies to achieve the goal more effectively (Skewes et al., 2015). Thus, it would be important for us to understand how the interpersonal coordination pattern (IP vs. MP) modulates the coordination outcome and the related brain-to-brain connectivity.

The essential distinction between the IP and MP is the relative frequency with which the actions of interacting individuals occur—IP has the movement frequency ratio of 1:1 while MP has the movement frequency ratios of non 1:1. According to the frequency-locking dynamics model, higher order frequency ratios were more difficult for individuals to perform or maintain than lower order frequency ratios (Treffner and Turvey, 1993; Peper et al., 1995). Related studies have provided evidence that when the frequency differences increased, the coordination of the group became less stable (Zhang et al., 2018), and more challenging (Gorman et al., 2017), which suggested that the MP would harm the coordination performance. However, it was found that team performance could also benefit from the asymmetry interpersonal relations in some complex social interactions (Wallot et al., 2016). In order to complete interpersonal coordination, individuals would use available information to predict another person's actions and then adjust their own actions (Knoblich and Jordan, 2003; Vesper et al., 2013). During this process, individuals might spontaneously form specific coordination strategies, for example, one person led the task and the other one followed (“leader-follower” relationship) (Davidson and Good, 2002; Goebl and Palmer, 2009; Richardson et al., 2015), or two persons equally adapted their behavior to each other (“hyper-leaders/followers” relationship) (Konvalinka et al., 2010; Pecenka and Keller, 2011). In MP condition, individuals moved at different frequencies, so that the partner's action could serve as a reference for individuals to adjust their action, which could facilitate the development of specific strategies and improve the interpersonal coordination performance.

In order to better understand the neural activities during social interaction, researchers have proposed a “two-person neuroscience” approach to study the physiological basis of human social interaction (Hari and Kujala, 2009). By recording the brain activities of two or more interacting individuals (i.e., hyperscanning), related studies have demonstrated that social interactions are associated with enhanced interpersonal brain synchronization (IBS), as measured by EEG (e.g., Jahng et al., 2017; Hu et al., 2018), fNIRS (e.g., Cui et al., 2012; Cheng et al., 2015; Liu et al., 2015; Hu et al., 2017), or fMRI (e.g., Schippers et al., 2010; Stolk et al., 2014). Specifically, the IBSs were found in various interpersonal coordination tasks. For example, studies have found inter-brain coherence when individuals moved at the same frequency, such as together key pressing (Cui et al., 2012; Cheng et al., 2015; Pan et al., 2017), coordinated group walking (Ikeda et al., 2017), and cooperative singing/humming (Osaka et al., 2014, 2015). Also, a related study has revealed synchronous oscillatory activities across individuals in a more complicated task in which a captain and a co-pilot coordinately operated the flight mission of plane takeoff and landing (Astolfi et al., 2012). By using a neuroimaging approach, studies have further observed the emergence of specific coordination strategies (e.g., leader-follower relationship) in terms of brain activities during individuals' interaction, demonstrating the greater information flow from the leader/sender to the follower/receiver (Schippers et al., 2010; Holper et al., 2012). Note that in these studies individuals were assigned with the dominant or secondary roles in the interaction tasks. If there are no such assigned roles, will individuals gradually form specific strategies during a continuous coordination activity? Moreover, will the brain-to-brain connectivity be varied by different coordination patterns?

Here, we explored how the coordination pattern (MP vs. IP) modulated the coordination outcome and the related brain-to-brain connectivity. We adopted a two-person complementary continuous joint drawing task to simulate the complex interpersonal coordination context in our real life. In the task, participants co-drew shapes of parallelograms on the computer by using a marker (one participant controlled the horizontal movement of the marker, while the other controlled the vertical movement of the marker), either at the same frequency (IP) or different frequencies (MP). Dyadic coordination performance in both IP and MP condition were calculated for comparison. Further, we recorded the brain activities from interacting individuals by simultaneously using the functional near-infrared spectroscopy (fNIRS) based hyperscanning technique. The prior region of interest was the frontopolar cortex, as it played an essential role in social interactions (especially in interpersonal coordination), where synchronous activities across brains were identified in previous fNIRS-based studies (Cheng et al., 2015; Nozawa et al., 2016; Ikeda et al., 2017). We would focus on the IBS during the interpersonal coordination. Further, Granger causality analysis (GCA) was used to provide a neurobiological suggestion of coupling directionality, i.e., which individual was more actively driving the other. In the study, we also collected participants' evaluations of self-control level, which was found to be related to the leadership (Fairhurst et al., 2014). Previous study has revealed that, compared to the followers, leaders would be generally associated with a stronger internal locus of control (self-control) and, therefore, a greater belief that outcomes were contingent on their behavior (Anderson and Schneier, 1978). Individuals' self-control bias could further modulate the degree of their adaptation to the partner when coordinating (Fairhurst et al., 2014). Collecting the participants' evaluations of self-control level and exploring their relationship with coordination performance and brain-to-brain connectivity would help us better understand the interpersonal coordination processes.

## Methods

### Participants

Sixty-two graduate and undergraduate students (age: 21.39 ± 2.36 years, 15 males) took part in this study. All participants were right-handed and had normal or corrected-to-normal vision. They were randomly assigned into pairs with an unacquainted partner, and then 31 dyads (15 female-male dyads and 16 female-female dyads) were created. All participants provided written informed consent before the experiment. Each participant would be compensated 40 *yuan* for his/her participation. The experimental procedures were approved by the University Committee on Human Research Protection of East China Normal University, and carried out in accordance with the recommendations in the Methods.

### Tasks and Procedures

**Figure 1:**
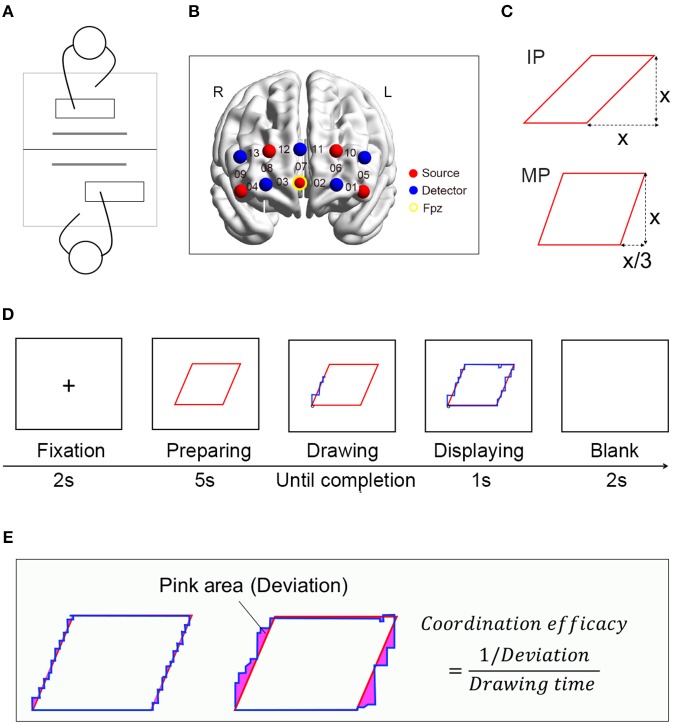
Experimental design for the coordination task. **(A)** Experimental setup. Participants co-drew shapes of parallelogram jointly. **(B)** Probe configuration. The integers on the cerebral cortex indicate the measuring channels. **(C)** Examples of target shapes for the IP and MP conditions. **(D)** Events and time flow in a drawing block. **(E)** The calculation for coordination performance (i.e., coordination efficacy). The deviation scores were determined by the amounts of pixels that traced shape deviated from the original shape (the pink area).

Two participants, randomly assigned as participant #1 and participant #2, were seated in front of a computer monitor separately (at the distance of 50 cm approximately), separated by a thick partition (Figure 1A). They would first have a 3-min resting phase, during which both of them were asked to relax and to remain still (Jiang et al., 2015). The resting phase served as the baseline in the current study.

Participants then performed a computerized complementary continuous joint drawing task adapted from the children's drawing game “Etch-a-Sketch” (Arueti et al., 2013; Gooijers et al., 2013). Their goal was to trace the lines of the target shapes on the screen (resolution: 1,920 × 1,080) by using a marker. The movement of the marker was controlled by two participants jointly, participant #1 controlled the horizontal movement (with the “A” and “S” keys) and participant #2 controlled the vertical movement (with the “↑” and “↓” keys). A total of eight drawing blocks were included in the task. Each drawing block began with a 2-s fixation (“+”) in the center of the screen, followed by a target shape in red (Figure 1D). The shape had the line width of 4 pixels. After 5 s, a blue marker (i.e., a filled circle with a diameter of 4 pixels) appeared randomly at one of the four vertexes of the target shape. Then participants could move the marker jointly by pressing specific keys (i.e., “A”/“S” and “↑”/“↓”). The gain of each key-pressing was set to 2 pixels. Participants were required to trace the lines of the shape precisely in a clockwise manner. When the marker went back to the start point, the drawing period ended. During the task, participants were not allowed to communicate with each other verbally. They could adjust their actions according to the real-time tracing path displayed on the screen.

Two coordination patterns (i.e., IP and MP) were arranged in the task. In the IP condition, the target shapes contained diagonal lines with the slope of 1 (the upper panel of Figure 1C); two participants needed to move at the same frequency to get better performance. In the MP condition, the target shapes contained diagonal lines with the slopes of 1/3 or 3 (the lower panel of Figure 1C). In this case, one participant (participant #1 or #2, randomly assigned) needed to move three times faster than the other one. Such a design was adopted in order to keep participants' actions as similar as possible across two conditions while the coordination pattern was manipulated. In MP condition, the participant who, relatively, moved a given drawing block faster would be regarded as the “higher-frequency participant,” and the other one as the “lower-frequency participant.” It was noted that an individual would possibly be the high-frequency participant in some drawing blocks and the low-frequency participant in others. Eight shapes (four in the IP condition and four in MP condition) used in the current study were equal in area, and their circumferences varied from 1,778 (MP condition) to 2,291 (IP condition) pixels. In the task, the eight shapes were presented in a random order.

Before the task, the participants completed a questionnaire of the locus of control (LOC) (Levenson, 1981). The questionnaire assessed the individual's general beliefs about the factors that have influenced one's own life. Three independent sub-scales included in the questionnaire measured the extent to which people believe that their lives are controlled by themselves (self-control), powerful others (other-control), or chance (chance-control). Each sub-scale included eight items. The questionnaire used a Likert response format (1–6 points, disagreement to agreement). In the current study, we mainly focused on the measurement of the self-control (Fairhurst et al., 2014).

### NIRS Data Acquisition

An ETG-7100 optical topography system (Hitachi Medical Corporation, Japan) was employed to simultaneously measure participants' concentrations of oxygenated hemoglobin (oxy-Hb) and deoxygenated hemoglobin (deoxy-Hb) during the experiment. The sampling rate was 10 Hz. Each participant had 2 × 5 probe patches (with a 3 cm distance between emitter probes and detector probes), forming a total of 13 recording channels (CHs). The patch was placed over the participant's forehead, covering the frontopolar cortex (FPC) and part of the dorsal lateral prefrontal cortex (DLPFC). The placement of the patch followed the International 10–20 system. The lowest probe row of the patch was aligned with the horizontal reference curve, with the middle optode located on the frontal pole midline point (Fpz) (see Figure 1B). Meanwhile, the middle probe column of patches was aligned along the sagittal reference curve. The correspondence between the NIRS channels and the measurement points on the cerebral cortex was determined using the virtual registration method (Lancaster et al., 2000; Tzourio-Mazoyer et al., 2002; Singh et al., 2005; Tsuzuki et al., 2007), which has been validated by a multi-subject study of anatomical craniocerebral correlation (Okamoto et al., 2004).

### Data Analyses

#### Behavior Performance

We recorded the coordinates of the marker's movements so that we could obtain dyads' tracing path by simple computation (Arueti et al., 2013). For each shape, we first calculated the deviation index of participants' drawings. It was determined by the number of pixels that the traced shape created by the participants (the blue line) deviated from the original shape (the red line) (Pink area, Figure 1E). For a given drawing block, the lower the deviation score indicated a more accurate precision by which the two participants drew. Since the circumferences of the eight shapes were not perfectly matched, which might have an impact on the deviation score, the original deviation scores were further divided by the circumference to eliminate the potential effect of the varying circumferences. The output deviation scores ranged between 0, representing a perfect score (flawless tracing), and tens of thousands, representing poor accuracy and a large deviation from the original shape. Given the inverse relationship between the deviation score and the accuracy performance, we took the reciprocal of deviation score as the accuracy performance. Lastly, accuracy performance per unit time, namely the coordination efficacy, was calculated as the behavior performance index in the current study. A high coordination efficacy value indicated a good coordination performance.

#### Self-Control Level

We calculated dyadic self-control level by averaging the rating scores of two participants according to previous studies (Bilek et al., 2015; Hu et al., 2017; Pan et al., 2017). Additionally, we calculated the differences between the two participants in self-control scores. Specifically, in a dyad, the participant who had a relatively higher self-control score was labeled as the high-self-control participant, while the other one was labeled as the low-self-control participant.

#### Interpersonal Brain Synchronization (IBS)

Both oxy-Hb and deoxy-Hb signals were automatically exported from the ETG-7100 system. Only the oxy-Hb signals were analyzed in the current study since the oxygenated hemoglobin is the most sensitive parameter of regional cerebral blood flow and provides the robust correlation with the BOLD signal (Hoshi, 2003). We employed the method of spline interpolation to detect the possible motion artifacts (Scholkmann et al., 2010) (by using the function hmrMotionArtifactByChannel from the Homer2 NIRS Processing package, Huppert et al., 2009). Then, the oxy-Hb data were high-pass filtered with the cut-off frequency of 0.01 Hz to remove longitudinal signal drift and noise from the instrument. After the preprocessing, the wavelet transform coherence (WTC) analysis of the two time series derived from two participants in dyad was conducted to assess the IBS for each dyad and each channel (Grinsted et al., 2004). The WTC toolbox used in this study was from Grinsted et al. (2004). According to previous studies (Cui et al., 2012; Hu et al., 2017), a larger coherence value would be observed when two persons interact, compared with that during the resting state. As participants continuously coordinated with each other, and they needed approximately 7 seconds to trace a single line, we mainly focused on the period from 3.2 to 12.8 s (frequency band: 0.08~0.31 Hz). Adopting this frequency band could also remove the high- and low-frequency noise as well. We then calculated the WTC values in our interested frequency band (i.e., 0.08~0.31 Hz) for each participant dyad during the resting phase and the drawing periods. Task-related IBS was defined as the increased IBS during the drawing periods compared to the resting phase (i.e., drawing period—resting phase). The false discovery rate (FDR) correction was applied for all 13 channels to control multiple comparisons, and the alpha-level was set to *p* < 0.05. Finally, the visualization of the IBS results was performed by BrainNet Viewer (Xia et al., 2013).

#### Directional Coupling

We conducted Granger causality analyses (GCA) to provide a neurobiological suggestion of coupling directionality, i.e., which individual was more actively driving the other, to explore whether there were specific coordination strategies developed during the interpersonal coordination. The Matlab Multivariate Granger Causality Toolbox (MVGC) was used to estimate the magnitude of Granger causality (i.e., GC) between two time series. GC is a statistical estimation of how much one time series is predicted by the history of another time series, taking into account how much it is predicted by its own previous history, in the form of a log-likelihood ratio (see more details in Barnett and Seth, 2014).

Our GCA was based on the preprocessed oxy-Hb signals during the drawing periods. Further, we converted the preprocessed signals into z-scores using the mean and the standard deviation of the signals recorded during the rest (baseline) session (Liu et al., 2015; Yang et al., 2016; Pan et al., 2018). In our study, we focused on the information flow between the high-self-control participant and the low-self-control participant (i.e., high-self-control participant → low-self-control participant, and low-self-control participant → high-self-control participant) in both MP and IP conditions. Additionally, we would also explore the information flow between the high-frequency participant and the low-frequency participant (i.e., high-frequency participant → low-frequency participant, and low-frequency participant → high-frequency participant) in only the MP condition, as there was no such a role of high- or low-frequency in the IP condition as two participants acted at the same frequency. Specifically, the GC was estimated at each direction for each dyad during MP and IP condition separately, and then one-sample *t*-tests were used to examine which direction differed from zero (Bonferroni adjusted). Independent-samples *t*-tests and repeated-measure ANOVA were further used to estimate the effects of direction and coordination pattern on the information flow.

## Results

### Behavioral Performance

**Figure 2:**
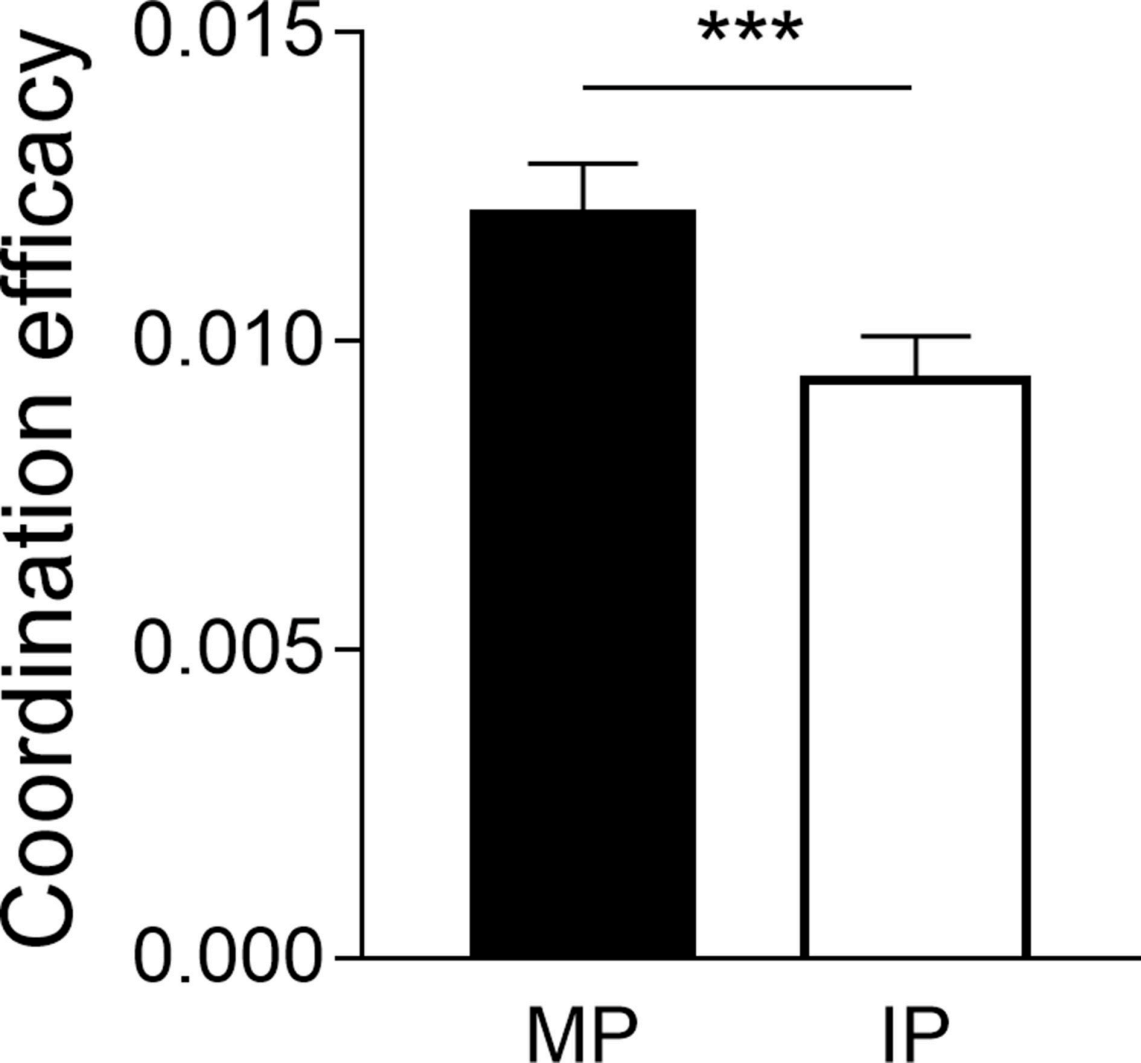
Behavioral performance. Better coordination performance was found in the MP condition compared to the IP condition. Error bars indicated standard errors. ***p < 0.001.

Participant dyads took approximately 27 s to complete drawing a shape. A pair-sample *t-*test was performed on the coordination performance, with the coordination pattern (MP vs. IP) as the within-subject independent variable. The result revealed a significant effect of coordination pattern, *t*_(30)_ = 4.53, *p* < 0.001, Cohen's *d* = 0.81, with the better coordination performance at the MP condition (0.0121 ± 0.0043) compared to the IP condition (0.0094 ± 0.0039) (Figure 2).

### The IBS During Interpersonal Coordination

**Figure 3:**
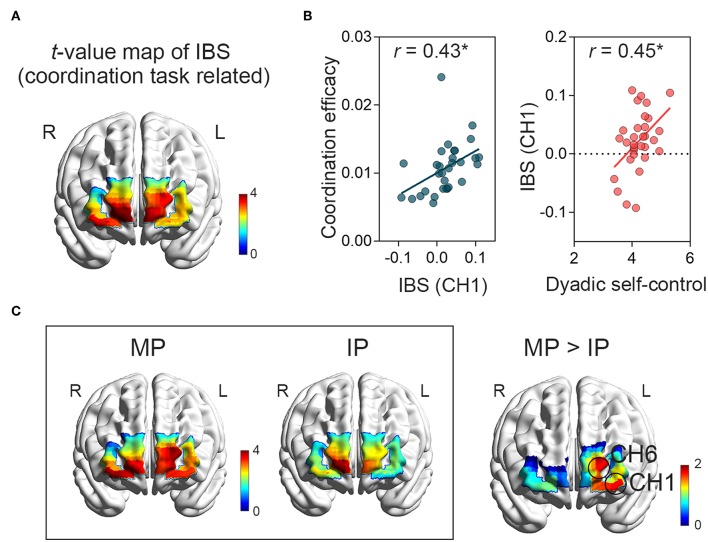
The IBS. **(A)** Dyads showed significant IBS at the bilateral FPC when coordinating. **(B)** The IBS at the left FPC (CH 1) was positively correlated with the coordination efficacy. Further, the detected IBS was related to dyadic self-control level. *p < 0.05. **(C)** The IBS in the MP condition tended to be greater than that in the IP condition at left FPC (CH 1).

We first examined whether there were increased task-related IBS during the interpersonal coordination. A series of one-sample *t*-tests were conducted on the task-related IBS for each channel. Channels 1~8 demonstrated significant task-related IBS (*t*s ≥ 2.24, *p*s < 0.05, Cohen's *d*s ≥ 0.40, Figure 3A) during interpersonal coordination. All the above channels except CH 5 were survived after FDR correction. These channels were located at the bilateral FPC. We further examined the relationship between the IBS and the coordination efficacy for each channel. However, only the IBS at CH 1, which was located at the left FPC, showed the correlation with the coordination efficacy, *r* = 0.43, *p* < 0.05 (Figure 3B, FDR uncorrelated). This channel had also displayed significant increased task-related IBS during the coordination tasks. These findings indicated that the bilateral FPC was generally engaging in interpersonal coordination, and specifically, the IBS at the left FPC predicted the coordination outcome. Further, the IBS at the CH 1 was closely related to the dyadic self-control level, *r* = 0.45, *p* < 0.05 (Figure 3B).

We then performed a series of one-sample *t*-tests on the task-related IBS for the two conditions separately to find the IBS elicited by the specific coordination pattern. For the MP coordination, significant IBS was found at CHs 1~7 (*t*s ≥ 2.20, *p*s < 0.05, Cohen's *d*s ≥ 0.39, Figure 3C), which were located at the bilateral FPC. Channels 1, 2, 3, 4, and 6 survived after FDR correction. Particularly, the IBS at the left FPC was associated with the coordination efficacy: CH 1, *r* = 0.47, *p* < 0.01. For the IP condition, significant IBS was found at CHs 2, 3, 4, 6, and 8 (*t*s ≥ 2.50, *p*s < 0.05, Cohen's *d*s ≥ 0.45, Figure 3C). The IBSs did not show their correlations with the coordination performance.

Direct comparisons between the IBS of these two coordination patterns were conducted. Pair-samples *t*-tests showed that the IBS in the MP condition tended to be greater than that in the IP condition at the left FPC (Figure 3C): CH 1, *t*_(30)_ = 1.75, *p* < 0.05 (one-tailed, FDR uncontrolled), Cohen's *d* = 0.31. CH 6, *t*_(30)_ = 1.78, *p* < 0.05 (one-tailed, FDR uncontrolled), Cohen's *d* = 0.32. However, no greater IBS was found in IP condition compared to the MP condition, *p*s > 0.05.

### Coupling Directionality

We carried out Granger causality analyses (GCA) to explore the directionality of the coupling during interpersonal coordination. As CH 1 was engaged in the interpersonal coordination performance, we would mainly focus on the signals of CH 1. The results showed that all directions being examined (i.e., “high-self-control participant → low-self-control participant” and “low-self-control participant → high-self-control participant” in both MP and IP conditions, as well as “high-frequency participant → low-frequency participant” and “low-frequency participant → high-frequency participant” in the MP condition) yielded significant increases in the GC relative to zero, *ps* < 0.05 (Bonferroni adjusted).

**Figure 4:**
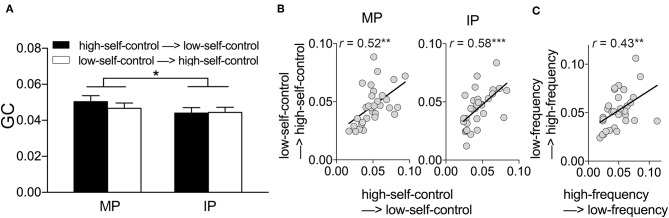
GCA results (CH 1). **(A)** Larger GC was found in the MP condition compared to the IP condition. Error bars indicate standard errors. *p < 0.05. **(B)** The GC from the high-self-control participant to the low-self-control participants was correlated with that from the low-self-control participant to the high-self-control participant in both the MP condition and IP condition. ***p < 0.001, **p < 0.01. **(C)** In MP condition, the GC from the high-frequency participant to the low-frequency participants was correlated with that from the low-frequency participant to the high-frequency participant. **p < 0.01.

Further, we performed a repeated measured ANOVA on the GC, with the direction (“high-self-control participant → low-self-control participant” and “low-self-control participant → high-self-control participant”) as the between-subject variable and the coordination pattern (MP vs. IP) as the within-subject variable. The results revealed a significant main effect of coordination pattern, *F*_(1, 60)_ = 4.14, *p* < 0.05, *η*^2^_partial_ = 0.06, with the larger GC in the MP condition (0.049 ± 0.005) compared to the IP condition (0.044 ± 0.004) (Figure 4A), suggesting the stronger interpersonal influence between coordinating individuals in the MP condition. However, we did not find a significant effect of the direction, *F*_(1, 60)_ = 0.19, *p* > 0.05, or the interaction between the direction and the coordination pattern, *F*_(1, 60)_ = 0.93, *p* > 0.05. Instead, we found a significantly positive correlation between the GCs of the two directions (Figure 4B), both in MP condition, *r* = 0.52, *p* < 0.01, and in the IP condition, *r* = 0.58, *p* < 0.001. Additionally, we examined the interpersonal influence between the high-frequency participant and low-frequency condition participant in the MP condition. The GCs of the two directions showed positive correlation (Figure 4C), *r* = 0.43, *p* < 0.01, and no evidence of coupling directionality was found, *t*_(60)_ = 0.54, *p* > 0.05. These findings indicated that two participants in dyad might not develop a leader-follower relationship during the coordination.

## Discussion

In the current study, we arranged a complementary continuous joint drawing task in both MP and IP conditions and measured the brain activities of the coordinating individuals simultaneously. We found better coordination performance, as well as the relatively greater task-related IBS, in the MP condition compared to the IP condition. GCA analyses further revealed that there were mutually interpersonal influences (i.e., information flow from one participant to the other) between coordinating individual. Such a kind of interpersonal influence was generally stronger in the MP condition compared to the IP condition. Finally, the detected IBS had a linkage to the dyadic self-control level. To our knowledge, this is the first fNIRS-based hyperscanning study directly exploring how interpersonal coordination pattern modulated coordination outcome and the related brain-to-brain connectivity.

In this study, significant IBS was observed during the interpersonal coordination at the FPC as expected. Specifically, we found the close relationship between the degree of increased IBS at the left PFC and the coordination efficacy. These findings highlighted the engagement of FPC in interpersonal coordination, which was consistent with the previous studies (Suda et al., 2010; Funane et al., 2011; Cheng et al., 2015; Ikeda et al., 2017). The measured frontopolar brain signals might indeed reflect functions involved in social interaction and coordination. The IBS at FPC induced by the interpersonal coordination could be possibly related to the temporally aligned recruitment of coordination-related cognitive processes, such as mentalizing and predicting each other's states (Frith and Frith, 2003; Amodio and Frith, 2006) and monitoring outcomes and maintaining meta-cognitive representations (Amodio and Frith, 2006; Rushworth et al., 2011).

The better coordination performance, as well as the relatively greater brain-to-brain connectivity (i.e., IBS and GC), was found in the multifrequency coordination pattern in the current study. It would be likely that the coordination between individuals might not simply follow the frequency-locking dynamics model that suggesting coordination performance would be damaged in the MP condition. Instead, MP settings might facilitate the information exchange between co-actors when they needed to perform quickly continuous movements and they could hardly see each other and were not allowed to communicate with each other directly. According to participants' informally oral reports (obtained after they completed the task), they generally felt difficult to maintain the actions, especially in the IP pattern condition as they could not well-predict their partner's action. In the MP condition, individuals did not need to move at the same time, so that they could observe and predict their partner's actions, and then adjust their own actions easily. Making correct predictions could be crucial for successfully performing joint actions (Vesper et al., 2013, 2017). Thus, the information exchange might be more efficient in the MP condition. The possibility was supported by the greater brain-to-brain connectivity, i.e., increased IBS and enhanced interpersonal information flow, during individuals' coordination in the MP condition. The brain-to-brain coupling between interacting individuals was found to mark the emergence of meaning and mutual understanding (Stolk et al., 2014). It was noted that participants' subjectively perceived different difficulty level in maintaining the two coordination patterns might also moderate the results to some extent; future studies could employ objective measurements or formal subjective ratings to clarify this issue.

With respect to the IP condition, we did not find significant correlations between the detected IBS (at CHs 2/3/4/6) and the coordination efficacy. It should be noted that interpersonal coordination activity generally involves multiple cognitive processes, such as mental representing, information sharing, and action monitoring (Vesper et al., 2017). It might be possible that the CHs (i.e., 2/3/4/6) were engaging in some cognitive processes during the interpersonal coordination, so that the IBS could be induced by the coordination activity but could not well-predict the overall degree of coordination efficacy. In the current study, only the IBS at CH 1 showed its correlation with the coordination efficacy during interpersonal coordination, which suggested that the IBS at CH 1 could be a predictor for the interpersonal coordination efficacy. We found the relationship of IBS at CH 1 and the coordination efficacy in the MP condition. Also, the IBS at CH 1 tended to show its correlation with the coordination efficacy in IP condition, *r* = 0.30, *p* < 0.05 (one-tailed). Given that in the IP condition the coordination efficacy was damaged, we speculate that the coordinating actions between two participants did not achieve success, so that the IBS at CH 1 did not reach a significant level, and the correlation of IBS and coordination efficacy was relatively weak.

During interpersonal coordination, two potential coordination strategies have been found in previous studies: the “leader-follower” relationship (Davidson and Good, 2002; Goebl and Palmer, 2009; Richardson et al., 2015) and the “hyper-leader/follower” relationship (Konvalinka et al., 2010; Pecenka and Keller, 2011). In the current study, we did not observe the difference in the GC between the related directions (e.g., “high-self-control participant → low-self-control participant” vs. “low-self-control participant → high-self-control participant”; “high-frequency participant → low-frequency participant” vs. “low-frequency participant → high-frequency participant”), indicating that individuals might not develop the primary or secondary roles. Instead, our GCA results showed the information flow from the high-self-control participant to the low-self-control participant was correlated with that from the low-self-control participant to the high-self-control participant, which suggested that the two participants influenced (or adapted) to each other mutually. Thus, we argued that participants performing the coordination task may form the “hyper-leader/follower” relationship in our study. Given that individuals could change their collaboration strategies and find a proper interaction mode to better achieve the goal (Skewes et al., 2015), it might also be possible that individuals were planning to form a “leader-follower” relationship at the beginning, and both of them happened to consider themselves being leaders or followers, forming a “hyper-leaders/followers” relationship. Future studies could further explore the development of coordination strategies during social interactions over time.

The present study pointed to a connection between the IBS during interpersonal coordination and the dyadic self-control level, which was consistent with the previous findings that the IBS was related to real-world social functioning (Bilek et al., 2015). Noted that we did not find a significant correlation between the self-control and the coordination efficacy (*r* = 0.15, *p* = 0.43), which suggested that the self-control level might modulate the interpersonal coordination processes but not directly contribute to the coordination outcome. The association between the IBS and self-control in the current study could reflect the mutual adaptation to some extent. We did not find the association between the detected IBS and two participants' difference in self-control. It indicated that the conception difference among individuals did not influence the IBS. These results provided additional evidence for a potential “hyper-leaders/followers” rather than “leader-follower” relationship between interacting individuals.

Several limitations should be addressed. First, only one frequency ratio (i.e., 1:3) was arranged in the MP condition in the present study. Related study has found that the in non 1:1 frequency ratios, the types of frequency ratios, such as integer (e.g., 1:3, 1:2) or not (e.g., 2:3, 3:5), could also affect coordination performance (Gooijers et al., 2013). Additional frequency ratios could be included in future studies. Besides, in the current task, two participants were not allowed to communicate with each other directly. Previous studies have revealed the roles of gaze or gesture communications in the joint action (Chen et al., 2013; Bilek et al., 2015). It would be worth exploring the effect of coordination pattern when two participants could see or hear each other. Second, we could not exclude the potential effect of task difficulty level on the establishment of IBS and coordination efficacy. Future studies could further manipulate or control the difficulty level of different coordination patterns. At last, we measured the FPC and DLPFC in the current study, however, other areas, such as right temporal parietal junction and superior frontal cortex, were also found to play an important role in social interaction (Cui et al., 2012; Liu et al., 2016; Pan et al., 2017). It could be possible that the significant IBS would be found in other regions in IP condition. The roles of the related brain regions during interpersonal coordination (including both MP and IP conditions) could be further examined by measuring the entire brain.

In summary, our work explored the effect of coordination pattern (MP vs. IP) on interpersonal coordination outcome and the related brain-to-brain connectivity. Compared to the IP condition, the MP condition could elicit better coordination performance and relatively greater brain-to-brain connectivity (i.e., IBS and interpersonal influence) between coordinating individuals. Finally, the IBS during the interpersonal coordination was related to dyadic self-control level. Taken together, our study revealed that the multifrequency pattern favors for the interpersonal coordination, which was associate with the enhanced brain-to-brain connectivity between coordinating individuals. These findings provide valuable insights for real-world teamwork, during which two or more individuals are required to coordinate in both space and time. Future studies can explore whether the effect is seen here with the bidirectional interaction also holds for the unidirectional interaction, as well as this effect in those with disorders of social cognition and behavior.

## Data Availability Statement

The datasets generated for this study are available on request to the corresponding author.

## Ethics Statement

This study was carried out in accordance with the recommendations of the University Committee on Human Research Protection of East China Normal University with written informed consent from all subjects. All subjects gave written informed consent in accordance with the Declaration of Helsinki. The protocol was approved by the University Committee on Human Research Protection of East China Normal University.

## Author Contributions

XC and YiH designed experiments. YP and YinH carried out experiments. XC, YiH, and YP analyzed experimental results. XC and YiH wrote the manuscript.

### Conflict of Interest

The authors declare that the research was conducted in the absence of any commercial or financial relationships that could be construed as a potential conflict of interest.
